# Hydrogel platforms for 3D cardiac tissue engineering and fibrotic disease modelling

**DOI:** 10.1002/2211-5463.70361

**Published:** 2026-10-21

**Authors:** Harrison Porritt, Annika Winbo, Jenny Malmström

**Affiliations:** ^1^ Department of Chemical and Materials Engineering, Faculty of Engineering and Design The University of Auckland New Zealand; ^2^ The MacDiarmid Institute for Advanced Materials and Nanotechnology Wellington New Zealand; ^3^ Department of Public Health and Clinical Medicine, Faculty of Medicine Umeå University Sweden

**Keywords:** cardiac tissue engineering, composite hydrogels, hydrogels, mechanotransduction

## Abstract

Tissue engineering offers significant potential to advance the understanding and treatment of cardiac disease by generating *in vitro* models that recapitulate myocardial physiology in both healthy and diseased states. Specifically, hydrogel‐based systems have been widely employed to engineer 3D cardiac tissues due to their ability to support cell viability and mimic aspects of the extracellular matrix. However, conventional hydrogels are inherently isotropic and electrically insulating, limiting their capacity to reproduce the anisotropic structure and conductive properties of native myocardium. To address these limitations, increasingly sophisticated hydrogel platforms have been developed that incorporate conductive, piezoelectric and/or aligned architectures to enhance electrical coupling, guide tissue organisation and promote cardiomyocyte maturation. These hydrogel‐based systems can also be tuned to recapitulate pathological states. Cardiac fibrosis is characterised by increased tissue stiffness, and researchers have sought to model this process by modulating the mechanical microenvironment to induce fibrotic phenotypes. With recently developed hydrogel platforms, mechanical properties can be controlled with both spatial and temporal precision, enabling systematic investigation of how dynamic and heterogeneous stiffness influences tissue formation, remodelling and disease progression. Although these platforms are not yet fully translational, they provide powerful tools to modulate multiple physiological parameters, thereby advancing our understanding of cardiac development and informing strategies to treat prevalent pathological conditions.

## Graphical abstract

Hydrogel‐based cardiac tissue models are advancing our understanding of heart health and disease. By incorporating electrical conductivity, aligned structures, and tunable mechanical properties, these platforms better replicate the complex environment of native heart tissue. Precise control of stiffness enables researchers to investigate cardiac fibrosis and tissue remodelling, providing new insights into disease progression and potential therapeutic strategies.

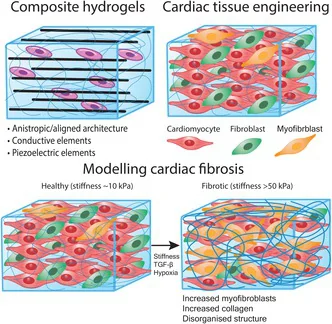

**Abbreviations**
CM: cardiomyocyte
CNT: carbon nanotubes
DBCO: dibenzocyclooctyne
DLP: digital light processing
DMD: digital micromirror device
DoF: degree of functionalisation
EB: embryoid bodies
ECM: extracellular matrix
GelMA: gelatin methacryloyl
GNR: gold nanorods
HA: hyaluronic acid
HAMA: hyaluronic acid methacrylate
HRP: horseradish peroxidase
iPSCs: induced pluripotent stem cells
MEW: melt electrowriting
MMP: matrix metalloproteinase
OMA: oxidised methacrylated alginate
PANi: polyaniline
PCL: poly(ε‐caprolactone)
PEDOT: poly(3,4‐ethylenedioxythiophene)
PEG: polyethylene glycol
PEGDA: poly(ethylene glycol) diacrylate
PLA: polylactic acid
PLGA: poly(lactic‐co‐glycolic acid)
PMGI: polymethylglutarimide
PPy: polypyrrole
PVA: polyvinyl alcohol
PVDF: poly(vinylidene fluoride)
PVDF‐TrFE: poly(vinylidene fluoride‐trifluoroethylene)
RU: ruthenium
TGF‐β: transforming growth factor beta
UV: ultraviolet
α‐SMA: α‐smooth muscle actin

Alterations in tissue mechanics are a defining feature of many pathological conditions, including Alzheimer's disease, osteoarthritis, kidney disease and chronic obstructive pulmonary disease [1]. In the heart, cardiac fibrosis represents a common pathological endpoint across diverse conditions, including chronic hypertension, myocardial infarction and hypertrophic cardiomyopathy [2]. Fibrotic remodelling is characterised by excessive extracellular matrix (ECM) deposition and progressive stiffening, with myocardial stiffness increasing from approximately 10 kPa in healthy conditions to 50–100 kPa under pathological conditions [3, 4]. The accumulation of fibrotic tissue can impair contractility and compliance, disrupt electrical conduction and ultimately contribute to heart failure [5].

Changes in the mechanical environment are not merely a consequence of fibrosis but can actively regulate cell behaviour through mechanotransductive signalling. In cardiomyocytes (CMs), pathways including integrin–FAK, RhoA/ROCK and YAP/TAZ are involved in sensing mechanical cues and regulating cytoskeletal organisation and maturation [6, 7]. The sarcomeric protein titin additionally functions as a molecular spring and mechanosensor, with developmental switching from the more compliant N2BA isoform towards the stiffer N2B isoform contributing to increased passive stiffness and maturation of contractile function [8, 9]. Similarly, increased matrix stiffness promotes cardiac fibroblast activation through mechanosensitive pathways, including YAP/TAZ signalling, which can interact with profibrotic TGF‐β signalling to promote myofibroblast differentiation and ECM deposition [10, 11]. Thus, pathological stiffening may establish a feed‐forward mechanism in which ECM remodelling alters cellular behaviour, further promoting fibrotic progression.

Current clinical interventions primarily focus on reducing cardiac workload, for example, through antihypertensive therapies, rather than directly reversing pathological ECM remodelling or restoring functional CMs within the affected region [12]. This limitation reflects an incomplete understanding of how mechanical cues regulate cellular behaviour during fibrosis progression. It highlights a critical need for *in vitro* cardiac models that faithfully recapitulate disease‐relevant mechanical microenvironments. In particular, platforms that enable precise control over matrix stiffness are essential for dissecting the role of mechanical signalling in fibroblast activation and evaluating emerging antifibrotic therapies [13].

Tissue engineering integrates cell biology, materials science and engineering to create functional *in vitro* human tissues. The advent of induced pluripotent stem cells (iPSCs) and robust CM differentiation protocols has enabled the engineering of human cardiac tissue *in vitro*, accelerating a shift away from traditional rodent models [14], which are constrained by ethical considerations and species‐specific differences, towards human‐relevant tissue constructs [15]. Within these platforms, disease modelling seeks to reconstruct pathological microenvironments that capture key aspects of disease initiation and progression. Disease phenotypes can be induced through several pathways, including CRISPR‐mediated genetic modification [16, 17] and the modulation of biochemical [18] and mechanical cues [19].

In this review, we evaluate current hydrogel‐based strategies for engineering 3D human cardiac tissues, with a particular focus on systems that provide control over the hydrogel's mechanical and structural properties. We further discuss their potential to enable next‐generation *in vitro* models that more faithfully recapitulate cardiac fibrosis progression and its defining hallmarks. Collectively, these advances are poised to accelerate the development of predictive disease models and improve the preclinical screening of antifibrotic therapies.

## Overview of 3D *in vitro* human cardiac models

Early *in vitro* cardiac models utilised iPSC‐derived CMs (iPSC‐CMs) cultured as 2D monolayers. Current differentiation protocols recapitulate key stages of early embryonic development, progressing through mesoderm induction, cardiogenic patterning, cardiac mesoderm specification and CM maturation [20]. Over the past decade, temporal modulation of Wnt/β‐catenin signalling has emerged as the dominant strategy [21]. One such protocol, the GiWi (GSK3 inhibition–Wnt inhibition) protocol, induces cardiac differentiation through sequential Wnt modulation, typically using the GSK3 inhibitor CHIR99021 to promote mesoderm formation, followed by Wnt inhibition with IWP2 or IWR1 to induce cardiac specification [21]. This strategy yields large amounts of immature, fetal‐like ventricular CMs that exhibit spontaneous contractility but lack the structural, metabolic and electrophysiological properties of mature CMs [20, 21, 22, 23]. Refinements have enabled partial subtype specification; for example, retinoic acid supplementation promotes atrial identity [24, 25, 26], while late‐stage reactivation of Wnt signalling can enhance differentiation towards nodal‐like pacemaker CMs [27, 28].

To produce more mature iPSC‐CMs, electromechanical stimulation strategies have been designed to enhance maturation. Electrical pacing (1–6 Hz) improves cellular alignment, intercalated disc formation and gap junction expression, while mechanical loading promotes force generation, myofibrillar organisation and the development of cell–cell junctions [29, 30]. Combining these cues yields the most mature phenotypes, as demonstrated by the Biowire II platform, which produces adult‐like characteristics, including T‐tubule formation, improved contractile function and inotropic responsiveness [31].

Early 2D monolayer systems provided a controlled platform for investigating CM behaviour. However, their physiological relevance is limited by the absence of 3D architecture, multicellular interactions and ECM cues, which are essential for cardiac tissue organisation and function [32]. To overcome these limitations, bioengineering strategies have increased model complexity by incorporating key structural and cellular features of the native myocardium.

Co‐culture models combine CMs with other cardiac cell types to better reproduce the cellular composition of native myocardium. Fibroblasts represent a substantial nonmyocyte population within the heart and play key roles in ECM deposition and fibrotic remodelling. This percentage of fibroblast is highly contested and depends on the cardiac region and method of cell identification, with recent estimates varying from 32% [33] to 64% [34]. Therefore, fibroblasts are frequently incorporated alongside endothelial and stromal cells to introduce paracrine signalling and cell–cell interactions. The inclusion of nonmyocyte populations has been shown to significantly increase CM maturation and to improve functional performance, including enhanced electrical coupling, increased contractile force generation and greater long‐term tissue stability [25, 35, 36, 37, 38].

Cardiac models have progressively transitioned from 2D cultures to 3D systems that more closely replicate the mechanical and structural cues cells experience *in vivo*. These systems enable physiologically relevant cell–cell and cell–matrix interactions, promote tissue‐level organisation and enhance CM maturation [32]. Broadly, engineered 3D models can be classified into scaffold‐free systems, which rely on the self‐organisation of cells, and scaffold‐based platforms that employ biomaterials to provide structural support and guide the formation of tissue‐like architectures.

### Scaffold‐free techniques

Scaffold‐free 3D cardiac organoids are generated by the self‐assembly of cells into multicellular aggregates, such as embryoid bodies (EBs) and cardiac spheroids. These systems exploit the cells' intrinsic capacity to adhere and organise without the need for exogenous scaffolds. Aggregate formation is achieved using nonadherent culture strategies, such as hanging drop cultures [39, 40], rotating suspension cultures [41, 42], nonadhesive moulds [43] or low‐attachment plates (forced floating) [44, 45].

EBs are 3D aggregates formed from pluripotent stem cells that can be directed towards cardiac lineages through staged differentiation [42, 46]. The most advanced EB‐based cardiac models employ modified versions of the GiWi protocols with the addition of BMP4 and Activin A during mesoderm induction [47] or BMP, VEGF and FGF during cardiac specification [48] to generate organoids containing diverse cardiac cell populations, including CMs, fibroblasts and endothelial cells. This multi‐lineage differentiation more closely reflects the coordinated development of native myocardium.

A key advantage of differentiating cells within 3D aggregates is the emergence of intrinsic gradients of oxygen, nutrients and mechanical tension. These self‐organised gradients regulate cell fate decisions, promoting spatially heterogeneous lineage specification and tissue organisation that more closely mimic early embryonic development than 2D systems [49]. As a result, such models can develop internal myocardial‐like cavities and spatially organised cellular compartments, recapitulating key features of *in vivo* tissue organisation [47, 50, 51]. In some cases, these spheroid or organoid systems form layered structures resembling endocardial‐, myocardial‐ and epicardial‐like regions [51, 52, 53].

In parallel, cardiac spheroids have emerged as a simpler scaffold‐free model. Unlike EBs, spheroids are formed from predifferentiated cells rather than iPSCs, enabling greater control over cell composition and improved reproducibility. For example, cardiac spheroids generated from monolayer‐differentiated human iPSC‐CMs have been produced either as pure CM populations [54] or as co‐cultures with supporting cell types such as fibroblasts or endothelial cells [29, 45, 55]. This bottom‐up assembly approach allows systematic investigation of cell–cell interactions and their contributions to tissue‐level function.

Despite their advantages, scaffold‐free systems offer limited control over mechanical properties and extracellular matrix composition, which are critical determinants of cardiac tissue function and fibrosis progression. This limitation has driven the development of scaffold‐based platforms, particularly hydrogel systems, that enable precise tuning of the cellular microenvironment.

### Scaffolded techniques

Scaffolded techniques involve encapsulating cells within biomaterial scaffolds to generate 3D constructs, most commonly using polymeric hydrogels. Hydrogels are water‐swollen, crosslinked networks that retain large volumes of water due to the presence of hydrophilic groups, such as carboxyl, amide, amino and hydroxyl moieties along the polymer backbone, mimicking the hydration and viscoelastic properties of native tissue, where glycosaminoglycans (GAGs) similarly regulate tissue hydration, mechanical compliance and force transmission [56].

Given that ECM plays a pivotal role in regulating cell survival, proliferation and function, many hydrogels are derived from natural or modified ECM components, including fibrin [57, 58], collagen [59], hyaluronic acid (HA) [60] and gelatin [61]. These polymers often present bioactive motifs such as arginine‐glycine‐aspartate (RGD) and Tyrosine‐Isoleucine‐Glycine‐Serine‐Arginine (YIGSR), which engage with integrins on the cell surface to promote adhesion, intracellular signalling and tissue organisation [62]. Other natural polysaccharide‐based materials, such as alginate [63] and chitosan [64], are commonly used but inherently lack intrinsic cell‐adhesive motifs. Despite their biological relevance, natural polymers can be limited by high batch‐to‐batch variability and the potential for nonspecific molecular interactions [65].

To address these limitations, synthetic hydrogels have been developed from polymers such as polyethylene glycol (PEG) [66], polyvinyl alcohol (PVA) [67], polylactic acid (PLA) [68] and poly(lactic‐co‐glycolic acid) (PLGA) [69]. These materials provide precise control over physicochemical properties, including stiffness, degradation kinetics and network architecture, but generally lack bioactivity and therefore require functionalisation with cell‐adhesive ligands to support cell adhesion [70].

To generate stable hydrogels, polymer chains are interconnected via physical or chemical crosslinks, thereby preserving the 3D network structure. This crosslinking not only maintains the hydrogel's architecture but also allows mechanical properties to be finely tuned by adjusting the crosslink density. Physical crosslinking arises from reversible noncovalent interactions such as hydrogen bonding, ionic interactions or hydrophobic associations. For example, alginate forms ionically crosslinked networks when divalent cations (Ca^2+^) coordinate with negatively charged carboxylate groups along the polymer chain [71], while agarose and gelatin form gels through hydrogen bonding and chain entanglement [72]. Chemical crosslinking forms permanent covalent bonds between polymer strands, providing greater mechanical stability and resistance to degradation [73]. Polymers capable of chemical crosslinking typically contain reactive functional groups such as amine (–NH_2_), thiol (–SH), carboxyl (–COOH), hydroxyl (–OH) or vinyl (C=C) groups, and crosslinking can be initiated through mechanisms, including enzymatic reactions, click chemistry, Michael‐type addition and free‐radical polymerisation [74]. Illustrations of these physical and chemical crosslinking mechanisms are shown in Fig. 1.

**Fig. 1:**
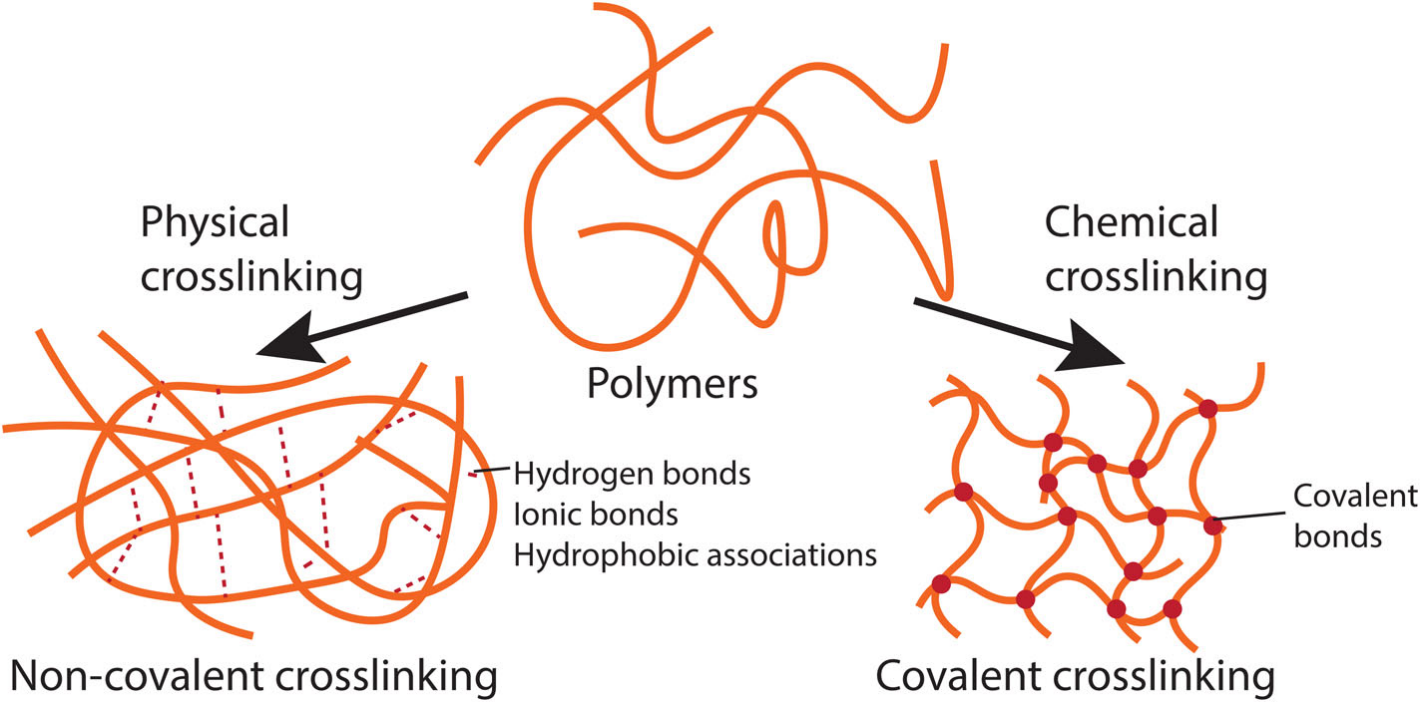
Illustration of polymers that undergo different forms of crosslinking to stabilise the polymer network. Either physical crosslinking which forms from noncovalent bonds such as hydrogen bonds, ionic bonds and hydrophobic associations, or chemical crosslinks which forms covalent bonds between polymer stands.

However, not all natural polymers possess functional groups compatible with crosslinking strategies. To overcome this limitation, polymers are often chemically modified or functionalised to introduce reactive groups that enable controlled crosslinking. For instance, polymers functionalised with thiol groups, such as thiolated PEG and HA, can participate in Michael addition or thiol‐ene click reactions to form crosslinks. Similarly, vinyl groups, such as those in methacrylates, are commonly grafted onto polymers, such as HA and gelatin, to enable free‐radical polymerisation. Methacrylated derivatives, such as HA methacrylate (HAMA) and gelatin methacrylate (GelMA) [75, 76], can be crosslinked via thermal, ultraviolet (UV) or visible‐light activation.

The application of hydrogels for 3D cardiac models was first demonstrated in 2002, when neonatal rat CMs were successfully encapsulated in fibrin gels [77]. Since then, hydrogel‐based systems have been widely adopted in cardiac tissue engineering to generate increasingly complex co‐culture models incorporating iPSC‐CM alongside cardiac fibroblasts, endothelial cells and smooth muscle cells [78]. Notably, iPSC‐CMs initially cultured in 2D monolayers and subsequently encapsulated in 3D gelatin‐based hydrogels showed improved organisation, sarcomere alignment and contractile protein expression compared with cells maintained solely in 2D culture over 50 days [79].

These findings have prompted the development of numerous engineered cardiac tissues by encapsulating cardiac cells within a variety of hydrogel systems, including GelMA [80, 81], gelatin–alginate [82], HA‐gelatin [83], Gellan gum‐gelatin [61], collagen‐Matrigel [30], PEG [84] and PEG‐fibrinogen hydrogels [85]. These scaffolded systems enable precise control over mechanical stiffness, cell–matrix interactions and biochemical cues, providing a versatile platform to model both healthy and diseased cardiac tissue.

## Optimising the hydrogel system for cardiac tissue

Native cardiac tissue exhibits a highly ordered, hierarchical architecture in which CMs are organised into aligned, laminar sheets that impart pronounced electrical and mechanical anisotropy [86]. This structural organisation is dictated by the ECM, whose aligned collagen fibre networks provide cues for CM orientation and elongation, which regulate proper electrical conduction and efficient myocardial contraction. However, most hydrogels are isotropic and electrically insulating, lacking the structural and conductive properties required to replicate native myocardial behaviour. To address these limitations, composite hydrogel systems have been developed that incorporate conductive, piezoelectric or structural components to enhance electrical signalling, provide anisotropic guidance and better recapitulate the native cardiac microenvironment.

### Conductive hydrogel systems

Conductive composite hydrogel systems are achieved through the incorporation of three major classes of materials into the hydrogel matrix: conductive polymers such as polypyrrole (PPy) [87, 88, 89], polyaniline (PANi) [90] and poly(3,4‐ethylene dioxythiophene) (PEDOT) [91, 92, 93]; carbon‐based nanomaterials, such as carbon nanotubes (CNT) [94, 95, 96, 97, 98, 99] and graphene oxide [100, 101, 102]; and metallic nanoparticles, including gold nanoparticles [103, 104] and gold nanowires [105, 106].

Among conductive polymers, PPy is one of the most extensively utilised due to its relative biocompatibility and ease of integration within hydrogel matrices, having previously been incorporated into HA [87, 107], collagen [88, 108, 109], PLA [110, 111] and alginate gels [112]. For example, incorporating 5 wt% PPy into GelMA–alginate hydrogels approximately doubled the electrical conductivity, increasing from 0.0615 to 0.124 Scm^−1^ [89]. A composite collagen–alginate hydrogel containing PEDOT:PSS significantly enhanced CM maturation, electrical coupling and expression of conduction‐associated genes compared with nonconductive gels, without any external electrical stimulation [113].

CNT‐incorporated GelMA hydrogels have been employed to engineer cardiac patches, wherein neonatal rat CMs exhibit enhanced structural organisation, more synchronous contraction and improved cell–cell coupling, Fig. 2A [97]. The incorporation of CNTs (up to 1 wt%) into collagen meshes showed no toxicity and enhanced CM adhesion and elongation [114]. Polydopamine‐modified reduced graphene oxide has been incorporated into GelMA hydrogels, which improve CM viability, alignment and synchronous beating, supporting the formation and functional maturation of engineered cardiac microtissues [101]. Metallic nanomaterials have also been incorporated into hydrogel systems; for example, gold nanoparticles [105] and conductive gold nanorods (GNR) [106, 115, 116]. GNRs coated with GelMA, which was then incorporated into GelMA/alginate bioinks, showed improved cell‐to‐cell coupling and promoted synchronised contraction, and higher contraction frequency, than GelMA/alginate bioinks Fig. 2B [117].

**Fig. 2:**
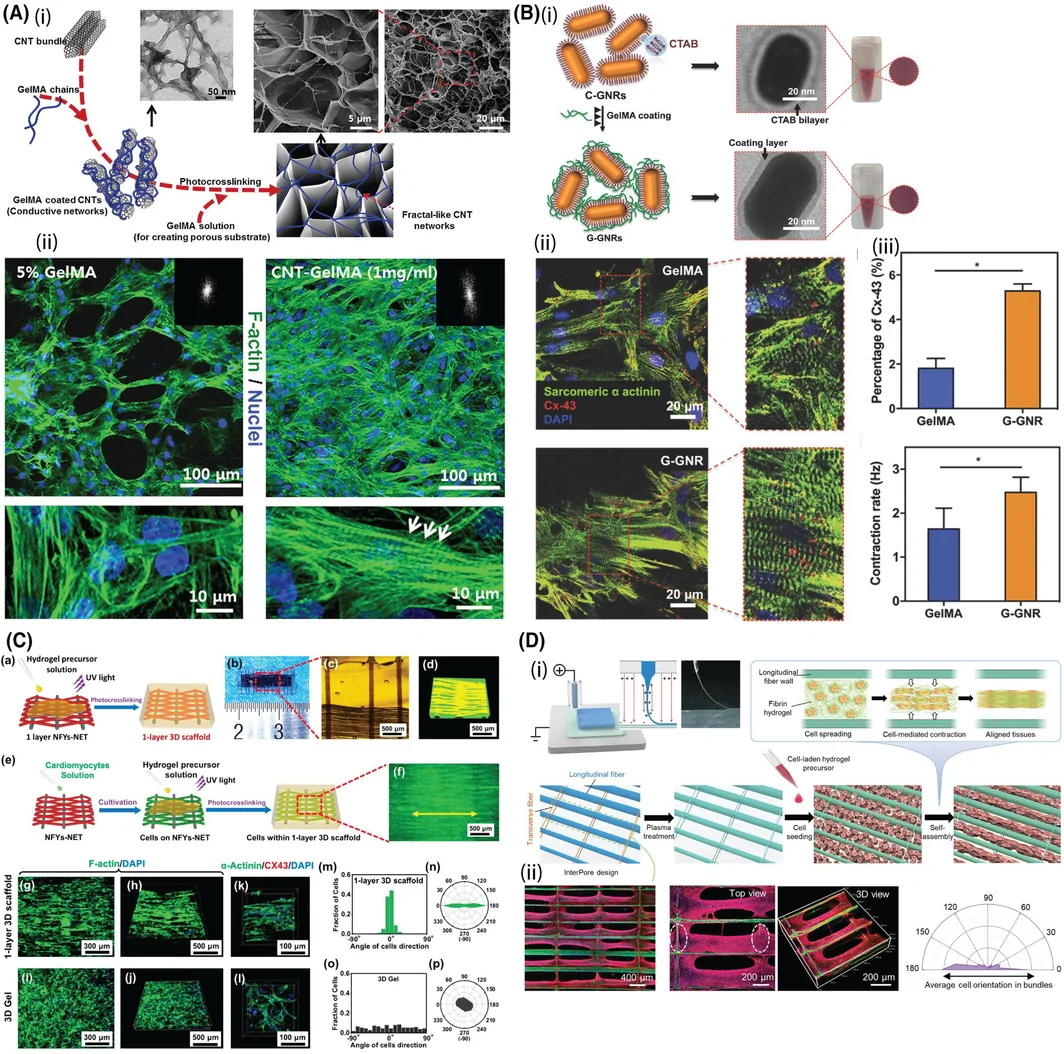
(A) (i) Transmission electron microscopy and scanning electron microscopy images of Gelatin methacryloyl (GelMA)‐coated carbon nanotube (CNT) fibres. (ii) Confocal images of cardiomyocytes (CMs) on pure 5% GelMA hydrogels and 1 mg·mL^−1^ CNT‐GelMA composites, demonstrating greater cell distribution and partial alignment of CMs, reprinted with permission from [97], copyright © American Chemical Society. (B) (i) Schematic of Gold nanorods (GNRs) coated with GelMA (G‐GNR). (ii) Confocal images of CMs cultured in GelMA/alginate and G‐GNR nanocomposite bioinks (0.1 mg·mL^−1^ G‐GNR, 7% GelMA, 2% alginate), stained for α‐actinin (green), nuclei (blue) and connexin‐43 (Cx‐43, red). (iii) Quantification of Cx‐43 expression (area coverage) and spontaneous beating rates; values were expressed as the mean ± the standard error (*P < 0.05). Reprinted with permission from [117], copyright © Wiley Advanced. (C) Schematic representation of aligned nanofibrous yarns (NFY) embedded within GelMA hydrogels containing CMs (A–E), alongside corresponding confocal images of CMs in NFY–GelMA constructs and pure GelMA controls (g–l), illustrating improved cellular alignment in NFY‐containing scaffolds (M–P). Reprinted with permission from [118] copyright © American Chemical Society. (D) (i) Schematic of engineered aligned cardiac tissue using an interpore microfibrous lattice fabricated via melt electrowriting (MEW) of aligned poly(ε‐caprolactone) (PCL) scaffolds, with fibrin hydrogels containing CMs deposited between fibres. (ii) Fluorescence image of CMs within aligned PCL–fibrin scaffolds stained for F‐actin (red), demonstrating pronounced cellular alignment. Reprinted with permission from [119], copyright © Wiley Advanced.

### Piezoelectric hydrogel systems

Piezoelectric hydrogel composites provide a strategy for introducing mechanoelectrical coupling into 3D models, whereby mechanical deformation is converted into local electrical signals. Piezoelectric polymers such as poly(vinylidene fluoride) (PVDF) and poly(vinylidene fluoride‐trifluoroethylene) (PVDF‐TrFE) can be incorporated as fibres or electrospun networks to generate electrical cues in response to mechanical loading [120, 121]. PVDF‐TrFE fibre scaffolds have supported alignment, spontaneous contraction and cardiac‐specific maturation of stem‐cell‐derived CMs [122, 123]. More recently, composite systems combining PVDF with conductive graphene have demonstrated simultaneous piezoelectric and electrical functionality, and showed enhanced structural organisation and expression of sarcomeric α‐actinin [124]. Systems incorporating PVDF and PCL microfibres, as well as magnetic PCL/Fe₃O₄ composites for externally controlled deformation, have been used to provide mechanical or piezoelectric stimulation. CMs cultured within these piezoelectric constructs and subjected to cyclic mechanical stimulation exhibited enhanced maturation, enhanced sarcomeric organisation and improved calcium handling, compared with nonpiezoelectric constructs [125].

Alternatively, piezoelectric ceramic nanoparticles, such as barium titanate (BaTiO3), can be incorporated into hydrogels [126, 127]. Polyethylene oxide (PEO)/silk fibroin/BaTiO_3_ composites have demonstrated piezoelectric properties and cytocompatibility with cardiac cells [128]. Piezoelectric materials may be interesting for fibrosis, as changes in the mechanical environment would offer additional changes to the electrical stimulus.

### Anisotropic hydrogel systems

To recapitulate the anisotropic architecture of native myocardium, early strategies focused on generating aligned fibrous scaffolds via electrospinning. Polymers such as poly(lactic‐co‐glycolic acid) (PLGA) [129], polymethylglutarimide (PMGI) [130] and poly(ε‐caprolactone) (PCL) [131] have been widely employed to produce aligned fibres that promote CM alignment. Although these studies were predominantly conducted in 2D, such fibrous architectures can be integrated with hydrogels to extend their structural guidance cues into 3D environments [132, 133]. For example, shear‐induced alignment of gelatin fibres within gelatin–alginate hydrogels was found to direct the self‐organisation of human CMs into anisotropic muscle‐like tissues compared with pure alginate hydrogels [134]. Similarly, aligned PCL/silk fibroin/carbon nanotube nanofibre yarns embedded within GelMA hydrogels enhanced CM elongation, alignment and maturation, Fig. 2C [118].

More advanced strategies have focused on engineering fibrous hydrogel composites with tunable architecture and mechanics. Systems incorporating fragmented PCL fibres within HAMA matrices enable independent control of fibre density and length and were found to regulate cellular contractility and promote the formation of anisotropic microtissues [135]. Large 3D‐printed anisotropic microfibrous lattices made from PCL via melt electrowriting (MEW), with CMs in fibrinogen seeded into the scaffold, guided CM alignment. These engineered tissues exhibit synchronous contraction, improved electrical connectivity and enhanced functional maturation compared with constructs in isotropic hydrogel environments, Fig. 2D [119]. In parallel, MEW produces highly ordered fibrous scaffolds of PCL incorporated in GelMA hydrogels with precise control over fibre architecture, which, when combined with hydrogels, promote CM alignment, anisotropic contraction and improved electrical propagation [136].

Recent advances in biofabrication have enabled the development of anisotropic hydrogels with properties beyond conventional fibre reinforcement. Filamented Light (FLight) biofabrication generates aligned fibrillar microgel structures within a bulk hydrogel via digital light processing (DLP) projection techniques, providing cues that direct CM elongation and organisation while simultaneously allowing tuning of matrix stiffness and degradability [137, 138, 139]. At higher spatial resolution, two‐photon polymerisation enables precise control of geometry at the nanometre scale [140]. Which can be done in hydrogel materials such as GelMA [141] or poly(*N*‐isopropylacrylamide) [142] to create complex structures. Complementary to these methods, granular hydrogels, formed by assembling many small microgel building blocks, create anisotropic structures by guiding the organisation of individual units [143, 144].

## Controlling the mechanical properties of hydrogels

The mechanical microenvironment plays a critical role in regulating cell development and differentiation through mechanotransduction pathways and plays a critical role in determining healthy and disease phenotypes. Recapitulating this dynamic stiffness range *in vitro* is therefore essential for modelling disease progression and interrogating mechanoresponsive pathways. Beyond absolute stiffness, emerging evidence highlights the importance of spatiotemporal heterogeneity, as native myocardium exhibits fibrosis that evolves dynamically and creates regional variations within the myocardium, necessitating model systems capable of both spatial patterning and temporal modulation of mechanics. Some systems fall outside the stiffness range of cardiac fibrosis but demonstrate approaches for tuning hydrogel mechanics.

Scaffold‐based hydrogels offer a distinct advantage over scaffold‐free systems by enabling precise and independent control of matrix mechanics, allowing researchers to investigate the effects of stiffness on cell phenotype and tissue development. The most direct approach to modulating stiffness is through polymer concentration. For example, increasing GelMA concentration from 5% to 15% wt/v increases the Young's modulus from 1.2 to 41.8 kPa [145]. Similarly, increasing methacrylated HA from 1% to 5% increases the Young's modulus from 3.5 to 24 kPa [146]. However, this approach simultaneously alters other matrix properties, including porosity and diffusivity, potentially confounding biological interpretation.

A more versatile strategy involves modulating the crosslink density, which governs the number of network junctions within the polymer matrix. Increasing crosslink density restricts polymer chain mobility, leading to higher stiffness and reduced swelling [76]. Crosslink density can be tuned through the number of covalent or physical bonds formed between polymer chains. For functionalised polymers such as GelMA or HAMA, the degree of functionalisation (DoF) directly determines crosslinking potential; increasing GelMA DoF from 28% to 91% at 10%wt raises Young's modulus from 3.8 to 29.9 kPa [147]. Crosslink density may also be controlled by altering the crosslinking stimulus. In ionically crosslinked alginate, increasing Ca^2+^ concentration from 21.8 mm to 40.5 mm raises stiffness from 11 to 21 kPa [91]. Similarly, in enzymatically crosslinked silk fibroin hydrogels, increasing H_2_O_2_ concentration (0.3–2.4 mm) during horseradish peroxidase (HRP)‐mediated crosslinking enables tuning of stiffness across a range of ~ 0.1–1.5 kPa [148].

In light‐initiated/photocrosslinkable hydrogels, stiffness is governed by the number of free radicals generated during polymerisation, which can be tuned by adjusting photoinitiator concentration, as well as the duration and intensity of light exposure. For example, GelMA hydrogels (10%wt) exhibit a broad range of Young's modulus values, from 30 to 160 kPa, when irradiation time is varied between 15 s and 2 min [149], or 4 to 45 kPa (15 s to 3 min) [150]. Altering light intensity enabled tuning of the stiffness between 1 and 5 kPa [151], or 4 and 35 kPa [152]. Representative strategies for controlling hydrogel stiffness are summarised in Table 1.

**Table 1:** Summary of techniques used to control the Young's modulus of hydrogels.

Tunable parameter	Tunable parameter range	Polymer	Crosslinking mechanism/stimulus	Young's modulus (kPa)	References
Polymer concentration (weight per cent, %wt)	5–15 wt%	Gelatin methacryol (GELMA)	Visible light	1.2–41.8	[145]
	1–5 wt%	Hyaluronic acid methacryol (HAMA)	UV light	3.5–24	[146]
Degree of functionalisation (DOF)	28% to 91%	GelMA	Visible light	3.8–29.9	[147]
Initiator concentration	21.8–40.5 mm (Ca^2+^)	Alginate	Ionic crosslinking	11–21	[153]
	0.3–2.4 mm (H_2_O_2_)	Silk fibroin	Enzymatic (Horseradish peroxidase)	0.1–1.5	[148]
	5–50 mm (Eosin‐Y)	GelMA	Visible light	80–122	[149]
Crosslinking stimulus	15 s–2 min (Light duration)	GelMA	UV light	4–45	[150]
	(17.8–128) mW·cm^−2^ (light Intensity)	GelMA	Visible light	4–35	[152]
	42–121 °C (Temperature)	Agarose	Thermal gelation	8–30	[154]

In addition to elastic properties, the viscoelastic behaviour of hydrogels can be tuned by controlling the composition, density and reversibility of the crosslinking network. Incorporation of reversible physical or dynamic covalent crosslinks allows polymer chains to rearrange under sustained deformation, thereby promoting stress relaxation and energy dissipation. Alginate is particularly useful for independently tuning elastic and viscoelastic properties because its ionic crosslinks are reversible and can readily undergo network rearrangement. For example, reducing alginate molecular weight or incorporating PEG spacers has been shown to accelerate stress relaxation without substantially altering the initial Young's modulus [155, 156]. Similarly, interpenetrating networks of alginate‐collagen crosslinked via reversible ionic interactions and permanent covalent norbornene–tetrazine bonds [157]. By controlling the relative contributions of these reversible and permanent interactions, the stress‐relaxation behaviour of the hydrogels can be tuned. Alginate–gelatin hydrogels functionalised with dibenzocyclooctyne (DBCO) have also been developed, enabling the formation of covalent azide–DBCO crosslink [158]. Synthetic PEG hydrogels have similarly been designed with tunable ratios of permanent covalent azide–DBCO crosslinks and reversible DBCO‐mediated noncovalent interactions, allowing independent control over stiffness, stress relaxation and degradation [159]. Increasing the contribution of dynamic interactions promoted network rearrangement and accelerated stress relaxation.

### Spatial control of hydrogel mechanics

Mechanical gradients act as potent regulators of cell behaviour [160]. In the heart, regions between healthy myocardium and fibrotic scar tissue are characterised by spatial variations in stiffness, with reported gradients in Young's modulus on the order of ~ 10 kPa·mm^−1^ [161]. These gradients give rise to durotaxis, a mechanosensitive process by which cells preferentially migrate towards stiffer substrates. Cardiac fibroblasts, in particular, exhibit strong durotactic responses, enabling their directed infiltration into progressively stiffening, fibrotic regions.

The predominant strategy for engineering systems with spatially varying mechanical environments involves precise spatial control over the hydrogel's crosslink density. One strategy involved varying the DoF of GelMA across a single construct, which was implemented by using multiJet 3D printing to spatial deposit different ratios of high and low DOF GelMA, followed by uniform UV crosslinking with the photoinitaor igacure 2959, Fig. 3A [162].

**Fig. 3:**
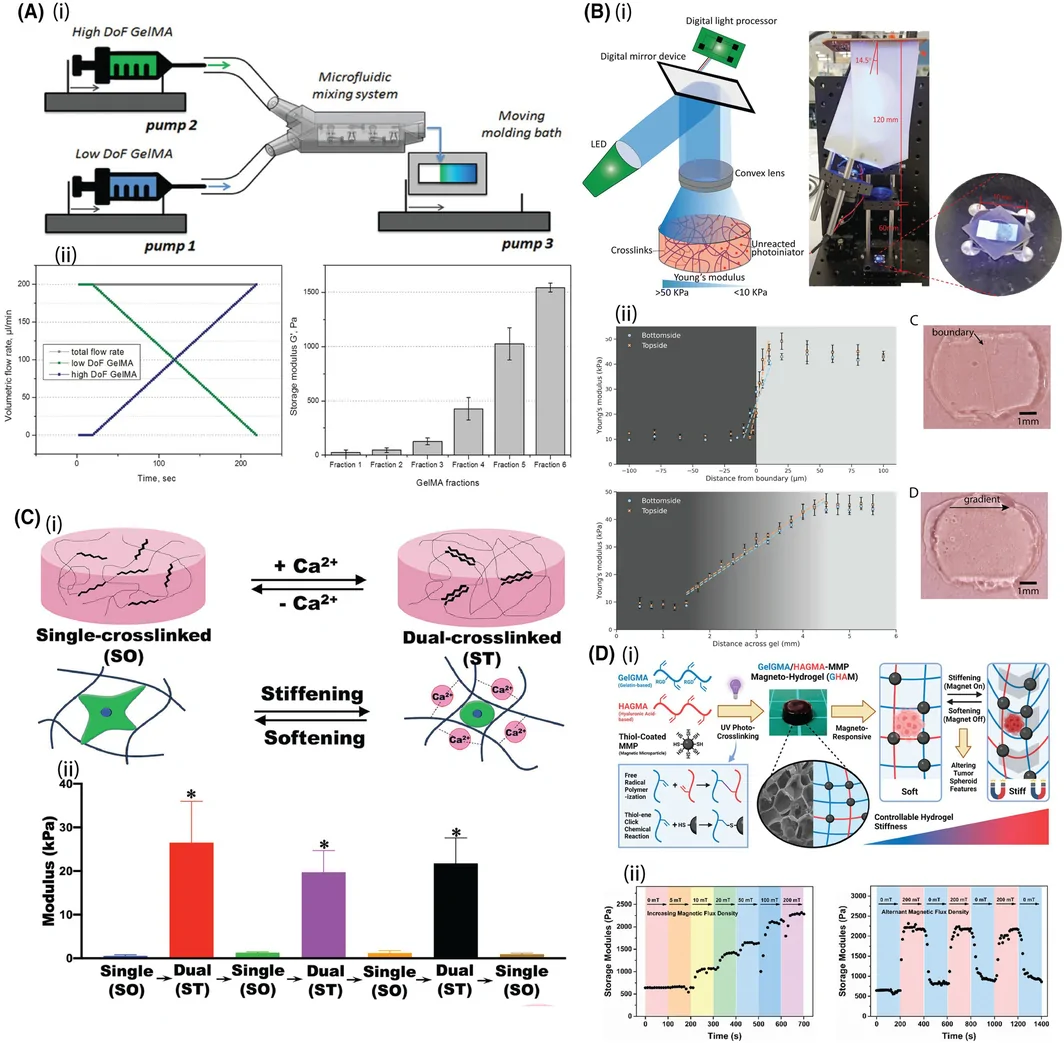
(A) (i) Schematic representation of a gradient fabrication system used to spatially control the Young's modulus through variation in the composition of low‐ and high‐degree‐of‐functionalisation (DOF) Gelatin methacryloyl (GelMA). (ii) Programmed volumetric flow profiles of the pumping system and resulting mechanical properties of GelMA gradient fractions, including storage modulus (G′) and loss modulus (G″), values expressed as the mean ± the standard error. Reprinted with permission from [162], copyright © under the Creative Commons CC‐BY licence. (B) (i) Schematic illustration of a digital light processing (DLP)‐based photopatterning system for hydrogel fabrication. (ii) Atomic force microscopy measurements of Young's modulus across the top and bottom surfaces of binary and gradient‐patterned hydrogels, values expressed as the mean ± the standard error. Reprinted with permission from [150], copyright © under the Creative Commons CC‐BY licence. (C) (i) Schematic of oxidised methacrylated alginate hydrogels incorporating both chemical and physical crosslinking mechanisms to modulate Young's modulus. (ii) Dynamic and reversible modulation of hydrogel stiffness, where stiffening was achieved by incubating single‐crosslinked (SO) hydrogels in 50× Ca^2+^ solution for 10 min and softening by incubating dual crosslinked (ST) hydrogels in 5× TE buffer (50 mm Tris, 5 mm EDTA, pH 7.5) for 10 min, values presented expressed as the mean ± the standard error (*P < 0.05). Reprinted with permission from [163], copyright © Elsevier. (D) (i) Schematic of magnetoresponsive hydrogels composed of gelatin–hyaluronic acid matrices embedded with magnetic microparticles. (ii) Hydrogel stiffness at varying magnetic flux densities and dynamic switching between approximately 500 and 2500 Pa as the magnetic field was alternated between 0 mT (magnet off) and 200 mT (magnet on). Reprinted with permission from [164] copyright © American Chemical Society.

More commonly, however, spatial variation in mechanical properties is achieved by modulating free‐radical generation during photopolymerisation. This has been achieved by varying the local concentration of the ruthenium (Ru) photoinitiator through diffusion‐driven distribution. Upon crosslinking, the Young's modulus of decellularised ECM varied linearly between 460 and 20 kPa over a distance of 3.5 mm, corresponding to a gradient of ~ 130 kPa·mm^−1^ [165]. This gradient is considerably steeper than the ~ 10 kPa·mm^−1^ [161]. Although the magnitude of a stiffness gradient does not necessarily determine the extent of durotaxis, studies comparing different gradient slopes have demonstrated that gradient magnitude can influence the cellular mechanisms underlying durotaxis [166]. For example, fibroblasts exhibited durotaxis across both shallow (~ 19 kPa·mm^−1^) and steep (~ 196 kPa·mm^−1^) gradients, but distinct actomyosin‐dependent responses were observed depending on gradient slope [166]. Thus, steep engineered gradients may be useful for investigating cellular responses to abrupt mechanical boundaries, although their biological effects may not directly reflect those occurring across physiological cardiac stiffness gradients.

Higher resolution in spatial patterning can be achieved by modulating the light intensity using projection‐based systems, such as DLP with digital micromirror devices (DMDs) [166, 167]. For instance, DLP‐based approaches have been used to generate stiffness ‘columns’ within GelMA hydrogels to promote cellular alignment, achieving stiffness variations of 1–5 kPa over ~ 25 μm (gradient = 160 kPa·mm^−1^) [151]. These systems also enable rapid pattern reconfiguration which was used to create sharp transitions in stiffness (e.g. 45–10 kPa over 15 μm, gradient ~ 2.3 MPa·mm^−1^) or physiologically relevant gradients over millimetre scales (~ 10 kPa·mm^−1^), These different spatial scales of stiffness variation provide opportunities to investigate cellular responses to both abrupt mechanical boundaries and more gradual stiffness transitions. Such gradients were used to control fibroblast mechanotransduction and migration, Fig. 3B [150].

### Geometric control of hydrogels

Photopatterning projection systems can also be used to control the geometry and architecture of hydrogels. Photocrosslinkable and photodegradable hydrogels can be patterned into defined microgrooves, channels and anisotropic structures, providing geometric cues that regulate CM morphology, alignment and organisation. Photodegradable GelMA has been patterned into microscale channels, with patterned substrates promoting CM alignment, cellular organisation and more regular beating compared with unpatterned hydrogels [168]. Similarly, micropatterned GelMA hydrogels containing iPSC‐CMs promoted cellular and F‐actin alignment, increased connexin‐43 and vinculin expression, longer sarcomeres and enhanced contractile anisotropy, with 20‐μm‐wide grooves separated by 20 μm providing the strongest contact‐guidance response [169]. Other micropatterned hydrogel approaches have likewise demonstrated that controlling feature geometry and cellular confinement can reproduce aspects of the anisotropic architecture of native myocardium and improve cardiac tissue organisation [170, 171]. Projection‐based approaches can extend this geometric control into 3D, for example, a DMD was used to directly bioprint GelMA containing CMs into defined unpatterned and parallel‐line architectures. CMs within the line‐patterned constructs exhibited preferential elongation and alignment along the printed geometry, whereas cells in unpatterned constructs showed less organised morphology and contractile directionality [172].

### Temporal control of hydrogel mechanics

Many tissues undergo dynamic, time‐dependent changes in mechanical properties during development, wound healing and disease progression. To recapitulate these processes *in vitro*, a range of temporally tunable hydrogel systems has been developed. In the context of cardiac tissue engineering, such dynamic control is particularly critical, as the myocardium undergoes mechanical remodelling during pathological progression.

Strategies to increase the Young's modulus at a defined time point typically rely on secondary crosslinking reactions, where additional crosslinks are introduced following initial gel formation. A simple example involves partial photopolymerisation of GelMA hydrogels, followed by a second irradiation step at a later time point (e.g. 24 h) to further increase crosslink density [173]. More advanced systems include dual‐network gels that can be crosslinked independently. For example, GelMA–alginate hydrogels were created, which are initially stabilised through ionic crosslinks within the alginate network. Subsequent photopolymerisation of the GelMA phase enables on‐demand stiffening of the matrix, increasing the Young's modulus from 1 to 18 kPa while maintaining high cell viability [174]. Similarly, PEG‐based hydrogels formed via thiol‐ene polymerisation can be postmodified by swelling in a solution containing additional multifunctional PEG macromers and photoinitiator, followed by a secondary light exposure to trigger further crosslinking, increasing the Young's modulus from 0.24 to 13 kPa [175].

Conversely, dynamic softening can be achieved through photodegradation or enzymatic cleavage. Photodegradation has become the most popular approach for this, with the majority of studies using o‐nitrobenzyl‐based photolabile linkers that degrade upon UV irradiation [176, 177, 178, 179]. One such example used an alginate‐polyacrylamide hydrogel whose mechanical stiffness can be significantly reduced by UV‐triggered photodegradation of o‐nitrobenzyl crosslinks. Initially, the hydrogels exhibit a Young's modulus of about 56 kPa, but after 6 h of UV irradiation, this decreases to ~ 33 kPa, and after 24 h to ~ 15 kPa [176]. Enzymatically degradable systems provide an alternative route to softening. Thiol‐ene crosslinked PEG hydrogels incorporating a dually degradable peptide linker containing both matrix metalloproteinase (MMP)‐ and thrombin‐sensitive cleavage sites can be enzymatically cleaved to reduce stiffness. The peptide functions as a crosslinker within the network, enabling both cell‐mediated degradation via MMP secretion and externally triggered softening through thrombin exposure. Cleavage of these linkers reduces crosslink density, resulting in controlled softening of the hydrogel matrix, reducing stiffness from 6.3 to 1.2 kPa [180].

Systems have also been developed to reversibly control hydrogel stiffness. For instance, oxidised methacrylated alginate (OMA), which combines photocrosslinking with reversible calcium ionic crosslinks, to reversibly control the stiffness from 0.5 kPa to stiff ~ 18–26 kPa. Stiffening is achieved by introducing Ca^2+^ ions to increase ionic crosslinking, while softening is induced by chelation (e.g. EDTA) to remove these crosslinks. This process can be repeated multiple times, allowing continuous switching between mechanical states, Fig. 3C [163]. In parallel, magnetoresponsive hydrogels composed of gelatin–hyaluronic acid matrices embedded with magnetic microparticles enable wireless, reversible control of stiffness under an external magnetic field, with reported modulation from 0.56 to 2.64 kPa, Fig. 3D [164]. Photoresponsive polyacrylamide–azobenzene hydrogels have been developed that can reversibly switch stiffness between 4 and 19 kPa under blue (450 nm) and green (525 nm) light. The system operates via light‐driven isomerisation of azobenzene moieties embedded in the polymer network. Upon visible‐light irradiation, azobenzene switches between an extended trans configuration and a bent cis configuration, thereby altering the effective length and orientation of the crosslinking elements [181].

## Previous fibrotic models

Cardiac fibrosis is the end stage of pathological remodelling, characterised by excessive deposition of ECM proteins and progressive myocardial stiffening, which disrupts native tissue architecture, impairs electrical conduction and reduces contractile function. Central to this process is the transition of quiescent fibroblasts into active myofibroblasts, which secrete large quantities of fibrillar ECM components, including collagen I and fibronectin, thereby reinforcing a profibrotic microenvironment. *In vitro*, a range of strategies has been developed to recapitulate these fibrotic phenotypes by inducing this fibroblast activation. This includes modulation of cellular composition, alteration of tissue Young's modulus, exposure to hypoxic conditions and biochemical stimulation.

Transforming growth factor (TGF)‐β remains the most widely employed biochemical inducer of fibrosis. For example, fibroblasts encapsulated in polysaccharide hydrogels with 2.5 nm TGF‐β exhibited pronounced myofibroblast activation and elevated collagen deposition [182]. Similarly, co‐culture systems, including CMs with fibroblasts in GelMA hydrogels (0.5 nm TGF‐β) [80], fibrin gels (10 nm TGF‐β), Fig. 4A [183] and cardiac spheroids (10 nm TGF‐β) [184] all demonstrated upregulation of myofibroblast markers [α‐smooth muscle actin (α‐SMA), fibronectin, collagen] and reduced CM contractile force in response to TGF‐β.

**Fig. 4:**
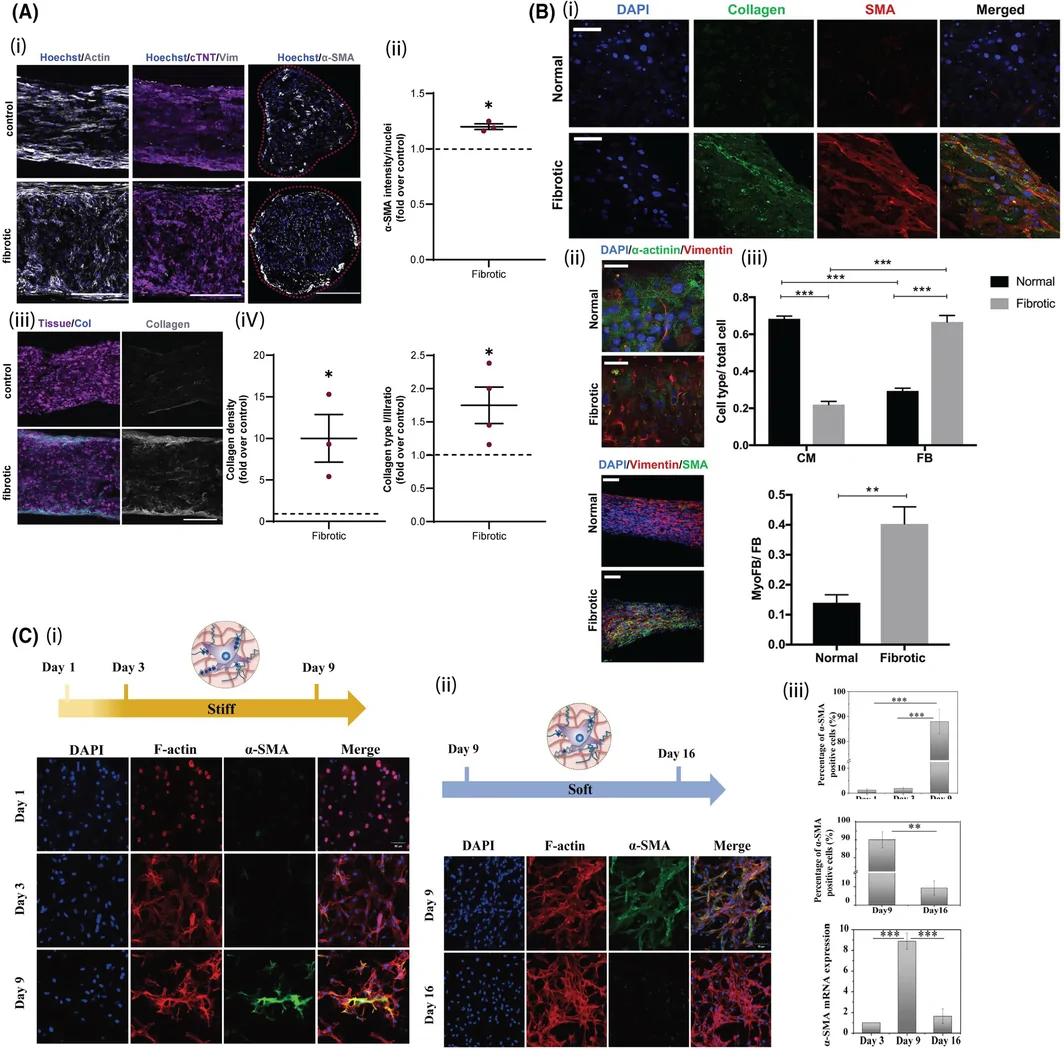
(A) Confocal images of induced pluripotent stem cell (iPSC) derived‐cardiomyocytes (CMs) and cardiac fibroblasts in fibrin gels with 10 nm transforming growth factor‐β1 (TGF‐β1) (fibrotic) and without TGF‐β1 control after 3 weeks, sections were stained for nuclei (Hoechst, blue) and α‐smooth muscle actin (α‐SMA) (grey). Red dashed line represents tissue outline. Scale bars: 200 μm. (ii) Quantification of α‐SMA staining. (iii) Control and fibrotic tissues Collagen (Col), cyan (left) and grey (right), and cells ‐ magenta (auto‐fluorescence). Scale bars: 250 μm. (iV) Area of collagen over tissue area and collagen ratio vs. control (dashed line), values presented as mean ± the standard error (*P < 0.05), reprinted with permission from [183], copyright © Elsevier. (B) (i‐ii) Confocal images of iPSC‐CMs and cardiac fibroblasts encapsulated within fibrin hydrogels at CM: Fibroblast ratios of 3 : 1 (Healthy) and 1 : 3 (Fibrotic), respectively, at a total cell density of 55 million cells·mL^−1^. Scale bars: 50 μm. (Iii) Quantification of CMs and cardiac fibroblast populations normalised to total cell number (mean ± SD, *n* ≥ 3, two‐way ANOVA), together with the proportion of myofibroblasts within the total fibroblast population (mean ± SD, *n* = 3, Student's *t*‐test) (*P < 0.05, **P < 0.01 and ***P < 0.001), Reprinted with permission from [185] Copyright under the Creative Commons CC‐BY licence. (C) Cardiac fibroblasts encapsulated within collagen–alginate hydrogels capable of dynamic and reversible stiffness modulation. (i) Hydrogel stiffening from 1 to 20 kPa was induced on Day 3 through the addition of 100 mm CaCl_2_. (ii) Subsequent softening from 20 to 1 kPa on Day 9 following treatment with sodium citrate. (iii) Quantification of α‐SMA expression during dynamic stiffening and softening, values presented as mean ± standard deviation comparisons performed using one‐way ANOVA followed by a *t*‐test (*P < 0.05, **P < 0.01 and ***P < 0.001). Reprinted with permission from [186] copyright © under the Creative Commons CC‐BY licence.

Physiologically relevant injury‐mimicking conditions, such as hypoxia and neurohormonal signalling, have been used to induce fibrosis‐like states. Multicellular organoids containing human iPSC‐CMs, fibroblasts, endothelial cells and adipose‐derived stem cells exposed to reduced oxygen tension (10% O_2_) and norepinephrine recapitulate several hallmarks of pathological remodelling, including increased fibroblast activation, increased expression of fibrosis‐related genes and elevated tissue stiffness [187]. Similarly, CM death, initiated by using cobalt chloride (CoCl_2_), showed an upregulation of fibrosis‐related genes in cardiac spheroids [48].

Alterations in cellular composition provide another powerful approach to modelling fibrosis. During pathological remodelling, fibroblast populations expand and dominate the cellular milieu, contributing to excessive ECM deposition and paracrine signalling through factors such as TGF‐β and angiotensin II. Increasing the fibroblast proportion beyond 50% has been shown to elevate collagen I deposition, reduce CM contractile force and decrease beating frequency in engineered cardiac microtissues [188]. In a fibrin‐based biowire platform, varying CM:fibroblast from 3 : 1 (healthy) to 1 : 3 (fibrotic) leads to marked increases in tissue stiffness (from ~ 7.6 to ~ 61.1 kPa), alongside myofibroblast activation, disrupted myofibrillar organisation, altered connexin distribution, prolonged contraction kinetics and reduced contractile force, Fig. 4B [185].

Mechanical cues are equally potent regulators of fibroblast activation, acting through mechanotransductive pathways to reinforce and sustain the myofibroblast phenotype during fibrotic stiffening. This has been demonstrated across a range of *in vitro* systems in which fibroblasts are encapsulated within hydrogels of defined and tunable Young's modulus. Representative platforms include hybrid collagen–alginate systems (1.2–120 kPa [189], 1–20 kPa [186]), GelMA (10–45 kPa [150]) and PEG hydrogels (7–32 kPa [190] 6–41 kPa [191]), all of which have been shown to promote fibroblast‐to‐myofibroblast transition in a stiffness‐dependent manner.

A system that can undergo dynamical softening has been used to examine the reversal of the myofibroblast transition. In a 3D photodegradable PEG hydrogel, decreasing the Young's modulus from 32 to 7 kPa redirected valvular myofibroblasts towards a quiescent phenotype [191]. Cardiac fibroblasts were initially encapsulated in collagen–alginate hydrogels that could undergo dynamic stiffness switching in soft gels (1 kPa). After stiffening (~ 20 kPa), the gels showed initial development of α‐SMA, vinculin and paxillin. After gel softening to 1 kPa, reductions in the α‐SMA, vinculin and paxillin expression, Fig. 4C [186]. Some of these values used to investigate fibroblast activation are significantly lower than those of reported pathological conditions (50–100 kPa) [3, 4]. Fibroblast activation and myofibroblast transition can therefore occur at stiffnesses below those measured at the endpoint of fibrosis, providing important insight into the early stages of fibrosis and the progression towards mature scar tissue. In contrast, higher stiffness values that more closely reflect established fibrotic tissue may be better suited to investigating scar maintenance, persistent myofibroblast activation and fibrotic expansion into adjacent healthy myocardium.

Recently, complexity has been added to these models to examine how spatial and temporal changes in mechanical properties can be used to investigate fibroblast response. Spatial gradients have also been implemented into hydrogel systems to investigate fibroblast migration, which occurs *in vivo* during early remodelling. Fibroblasts encapsulated in stiffness‐patterned GelMA hydrogels showed stiffness‐dependent fibroblast‐to‐myofibroblast transition and migrated along gradients of 12 kPa·mm^−1^ towards stiffer regions, and also aligned ±45 degrees parallel to the gradient [150]. This has also been observed in collagen gels [192].

Viscoelastic properties can also influence both CM and fibroblast behaviour, providing mechanical cues that are not captured by stiffness alone [193]. Failing myocardial tissue is more resistant to deformation and exhibits slower stress relaxation [194]. A 3D cardiac spheroid model of fibrosis by varying the ratio of CMs to cardiac fibroblasts demonstrated that increased fibroblast content led to greater collagen deposition and altered tissue rheology. AFM‐based stress‐relaxation measurements showed that fibrotic spheroids exhibited faster stress relaxation, highlighting that cardiac fibrosis alters not only tissue stiffness but also its time‐dependent viscoelastic properties [195]. Double‐crosslinked alginate–gelatin hydrogels functionalised with DBCO allow for varying the stress relaxation properties with minimal change in elastic modulus. Softer stress‐relaxing matrices promoted cell spreading, whereas stiffer stress‐relaxing matrices enhanced asymmetric cell elongation in cardiac fibroblasts [158].

Micropillar platforms provide a complementary approach to stiffness‐controlled hydrogel models by incorporating mechanically defined boundary conditions for contracting cardiac tissues. In these systems, CMs and fibroblasts self‐organise and compact around flexible posts or micropillars. Variations in pillar stiffness and geometry can therefore alter the mechanical resistance experienced by the tissue [196, 197, 198]. DLP 3D‐printed hydrogel micropillar made from poly(ethylene glycol) diacrylate (PEGDA) with tunable geometry and stiffness was used to support iPSC‐CMs embedding in collagen‐matrigel hydrogels. The micropillars aided in CM maturation and alignment compared with conventional 2D culture. Increasing micropillar stiffness from 2 to 27 μN·μm^−1^ showed irregular sarcomere organisation, increased expression of pathology‐associated genes and BNP secretion [199]. Similarly, polydimethylsiloxane (PDMS) posts increased maturation of CMs embedded in fibrin hydrogels. However, very high afterload also increased markers associated with pathological hypertrophy and fibrosis [200].

## Outlook and conclusion

Hydrogel‐based platforms have emerged as powerful tools for modelling cardiac tissue architecture, mechanics and function. These models have evolved from 2D iPSC‐CM monolayers to more complex composite hydrogel‐based systems, with significantly more tunable mechanical properties and a more complex structural architecture that captures some of the structural, electrical and temporal complexity of native myocardium. Despite this progress, key challenges remain before these models can be used as predictive patient‐specific models for modelling the effects of different drugs or treatments.

Future research will continue to add complexity to these models to further recapitulate structures/hallmarks for mature adult myocardium. This complexity will likely stem from integrating previously established techniques to increase electrical conductivity, structural anisotropy and dynamic tunability within a single platform. Emerging biofabrication technologies are poised to play a transformative role in this space. Incorporation of more advanced techniques into current hydrogel systems, such as DLP [201], two‐photon polymerisation [140], volumetric bioprinting [202] and melt electrowriting [203] would enable greater control of 3D architecture and mechanical properties across the micro‐ and macro‐scales, giving researchers more tools to mimic 3D myocardial architecture and multiple cell phenotypes/expression, bridging the gap between simplified *in vitro* models and native tissue complexity.

Current hydrogel systems are typically limited to small, thin constructs due to diffusion constraints, restricting their ability to model the layered architecture of native myocardium. This limitation becomes particularly important at high cell densities required to form a confluent tissue, where insufficient diffusion of oxygen and nutrients into the centre of the construct can lead to hypoxic or necrotic cores. Strategies to induce vascularisation will therefore be essential to enable the development of thicker, more physiologically relevant tissues composed of stacked cardiac laminae that better represent the myocardium, and to investigate the interactions between lamina sheets, and to determine how those interactions can be affected during fibrosis.

In parallel, improving the maturity and phenotypic stability of iPSC‐CMs remains a major challenge. Although 3D hydrogel systems enhance cellular organisation and functional output compared with 2D culture, iPSC‐CMs frequently retain an immature phenotype. Progress is likely to arise from advances in both 2D differentiation protocols and 3D maturation strategies, as well as from the direct differentiation of cells within 3D hydrogel environments, analogous to approaches used for embryoid body formation [47, 50, 51]. Incorporating biophysical cues during differentiation, including anisotropy, electrical stimulation and mechanical loading, may further promote structural organisation and functional maturation.

## Conflict of Interest

The authors declare no conflict of interest.

## Data Availability

No new data were generated or analysed in this review article.
